# Clinicopathological characteristics, treatments and oncological outcomes in metaplastic breast cancer: a Brazilian multicenter analysis

**DOI:** 10.3389/fonc.2025.1568178

**Published:** 2025-09-29

**Authors:** Talita Aparecida Riegas Mendes, Idam de Oliveira-Junior, Fabrício Palermo Brenelli, Cassio Cardoso-FIlho, Luiz Carlos Zeferino

**Affiliations:** ^1^ Department of Mastology and Breast Reconstruction, Barretos Cancer Hospital, São Paulo, Brazil; ^2^ Department of Tocogynecology, School of Medical Sciences (FCM), University of Campinas (UNICAMP), Campinas, Brazil; ^3^ Postgraduate Program of Tocogynecology, School of Medical Sciences (FCM), University of Campinas (UNICAMP), Campinas, Brazil

**Keywords:** breast carcinoma, breast neoplasm, metaplastic carcinoma, triple-negative carcinoma, metaplastic breast carcer

## Abstract

**Introduction:**

Metaplastic breast carcinoma (MBC) is a highly heterogenous group of tumors. MBC differs from other invasive carcinomas in clinical presentation, prognosis and response to treatment. The tumor is more aggressive and the most effective form of treatment is still uncertain for this patient population, given the particularities of the disease.

**Subjects and methods:**

This is a retrospective, descriptive study analyzing data from women admitted for MBC treatment to participating centers (Hospital de Amor, Barretos, and Center for Integral Attention to Women’s Health, CAISM/UNICAMP) between 2010 and 2020.

**Results:**

A total of 102 women with pathologically confirmed MBC and presenting non-metastatic disease were included. The average age at diagnosis was 53 years, 73.3% were triple-negative (TN) subtype and mean tumor size at diagnosis was 7.4 cm. We found that 59% of patients were clinical stage III at diagnosis and 82.3% of the cases underwent mastectomy. Despite the use of neoadjuvant treatment in 52.9% of patients, the pathological complete response (pCR) rate was only 7.4%. Around 46% of patients underwent adjuvant chemotherapy and 79.4% received adjuvant radiotherapy. We observed a 5-year overall survival (OS) of 59,7% and a 5-year disease-free survival (DFS) of 54.4%. Adjuvant chemotherapy, smaller tumor size and absence of lymph node disease were associated to better DFS and OS.

**Conclusion:**

MBC presented as a large nodular lesion at diagnosis, the most frequent metaplastic subtypes presented squamous and mesenchymal differentiation, almost 80% were triple-negative tumors, however, responses to neoadjuvant chemotherapy can be considered poor. A higher number of metastatic lymph nodes and larger tumor size were associated with worse DFS and OS, meanwhile the women who undergone to adjuvant chemotherapy showed better DFS and OS. Furthermore, most recurrences occurred in the first 24 months of follow-up, stabilizing at approximately 50% after 36 months, and most deaths occurred in the first 36 months, stabilizing thereafter, which is a clinical pattern of very aggressive tumors.

## 1 Introduction

Metaplastic breast carcinoma (MBC) accounts for 0.2% to 5% of all breast cancers and is a special histological subtype of invasive breast cancer that has a more aggressive behavior (1–4). The term “metaplastic carcinoma” was first described by Huvos et al. in 1973, as a breast carcinoma with epithelial and sarcomatous components and only from 2000 they were recognized by the World Health Organization (WHO) as a histological special subtype of breast cancer (5, 6).

MBCs are defined as a malignant mixture of glandular and nonglandular elements, with epithelial and/or mesenchymal components. Its morphology may present epithelial neoplastic differentiation, into squamous cells or into mesenchymal tissue (cartilage, muscles or bone). In 2019, the WHO classified MBCs according to their morphological characteristics into: adenosquamous carcinoma, squamous cell carcinoma, spindle cell carcinoma, fibromatosis-like metaplastic carcinoma with mesenchymal differentiation (e.g. chondroid, osseous, sarcomatous or neuroglial differentiation), and mixed MBC (7, 8). These tumors may also be classified as low-grade variants (adenosquamous and fibromatosis-like carcinoma) or high-grade variants (the others) (1, 7, 9).

Clinically, MBC often presents as a large, rapidly growing palpable mass in the breast, in postmenopausal women. Imaging studies may show changes similar to those of invasive ductal carcinoma (IDC) of the breast (3, 10, 11). Axillary lymph node involvement is lower than that expected for other types of breast cancer, particularly considering tumor size at the time of diagnosis (12, 13). The risk of tumor recurrence and distant metastasis appears to be higher than in other breast tumors, particularly for the lung, bones and central nervous system (4, 11, 14).

Although rare, this tumor has been increasingly diagnosed in the past years, especially due to better histopathology recognition (7). A particularity of MBC is that they present a triple-negative (TN) breast cancer phenotype in 77% to 89% of cases, which may be associated with the absence of extensive glandular components in these tumors (1, 7, 10, 13, 15). In addition, MBC are often large tumors at diagnosis and typically have a higher risk of hematogenic than lymphatic metastases (16, 17).

The most effective treatment for MBC remains uncertain and currently follows established therapies for management of IDC (13–17). Thus, being a heterogeneous disease, different responses to the available treatments can be observed. In this context, it is necessary to gather or confirm MBC information in Brazilian women, data that has not been evaluated until now, contributing to the adoption of more effective therapeutic strategies for more favorable oncological outcomes.

## 2 Subjects and methods

This is a retrospective descriptive study, with data collected from medical records of women admitted to two comprehensive cancer centers integrated into the Brazilian public healthcare system, Women’s Integral Healthcare Center - CAISM/UNICAMP (Campinas - São Paulo) and Hospital de Amor – Barretos (Barretos – São Paulo). These women included in the study were admitted for treatment from January 2010 to January 2020, and showed histopathological diagnosis of MBC by biopsy and/or surgical pathology report. Initial disease at clinical stage IV, history of previous cancer and lack of treatment data in patient medical records were criteria for exclusion.

Sociodemographic, clinical and pathologic features and therapeutic procedures were analyzed. The histopathological variables included, based on breast biopsy and/or surgical specimen, were the morphological subtype of MBC (according to the WHO-2019 classification), histological grade, molecular subtype (based on the immunohistochemical evaluation of estrogen and progesterone receptors and Her-2 expression), cellular proliferation index by ki67 expression, association with other types of breast carcinomas and number of involved lymph nodes. Treatment and outcome were assessed by the following variables: type of breast and axillary surgery, neoadjuvant chemotherapy, adjuvant chemotherapy, adjuvant radiotherapy, adjuvant endocrine therapy, clinical response (through preoperative clinical evaluation) and pathological response (through the RCB index – *Residual Cancer Burden*) after neoadjuvant chemotherapy. The treatment of the women was based on the current treatment guidelines of each institution at the time of admission, as well as patient follow-up. The disease-free survival rate was calculated based on the interval (in months) between the diagnosis and the first oncological event (locoregional recurrence, distant recurrence or new primary cancer) and overall survival was calculated based on the interval (in months) between diagnosis and death (all-cause mortality).Categorical variables were presented in absolute (n) and frequency (%). Numerical variables were presented as the mean, median and quartiles. To evaluate the association between categorical variables, the chi-square or Fisher’s exact test was used. For numerical variables, the Mann-Whitney test was used. Disease-free survival and overall survival curves were constructed by the Kaplan-Meier method and compared by the log-rank test. Cox regression analyses, univariate and multivariate analysis with Stepwise variable selection criteria, were used for analyzing disease-free survival and overall survival. The significance level adopted was 5% (p<0.05). The SAS System for Windows (Statistical Analysis System), version 9.4. SAS Institute Inc, 2002-2012, Cary, NC, USA was used.

This study was approved by the Research Ethics Committee (REC) of both participating centers, under CAAE 57430122.1.1001.5404 (Unicamp) and CAAE 57430122.1.2001.5437 (Hospital de Amor in Barretos). Recommendations of document n°146 (2021) of the Health Ministry and Resolution 466/2012 of the National Health Council (NHC) were followed.

## 3 Results

A total of 133 patients with a histopathological diagnosis of MBC were identified, with 10 patients excluded from the analyses due to a history of previous neoplasia, 20 due to being diagnosed at stage IV and one who did not continue treatment. Thus, 102 patients were included in the statistical analyses.

Clinical stage IV was the initial diagnosis in 16.4% of cases, and 55% (11/20 patients) of these had pulmonary metastases, isolated or concomitantly with other sites (lymph node, osseous and/or hepatic) (data not shown). **Table 1** show the descriptive characteristics of patients without metastatic disease at diagnosis. Mean patient age at diagnosis was 53 years (standard deviation of 13.9) and around 56.8% were diagnosed after 50 years of age. The breast lesion was identified as a nodule in most cases, with an average size of 7.4 cm, and the tumors were classified as clinical stage III in 59%, in an initial evaluation. Clinical lymph node involvement (cN+) occurred in 51% of the women and lymph node disease on surgical pathology (pN1, pN2 or pN3) was reported in 45% of the patients. Pathological lymph node status was not assessed in only one patient who underwent only a mastectomy. Histological grade 3 represented 95.5% of cases and, on immunohistochemistry, the most frequent molecular subtypes were triple-negative and luminal, in 73.3% and 18.8% of tumors, respectively. The mean ki67 was 65%, although this data was evaluated in only 88 of the 102 cases (**Supplementary Table 1**).

**Table 1:** Descriptive characteristics of the sample of patients with metaplastic breast carcinoma without metastatic disease.

Variable	*N* = 102 (%)
Hospital	
Center for Integral Attention to Women’s Health (CAISM/UNICAMP)	19 (18.6)
Hospital de Amor	83 (81.4)
Age	
<50 years	44 (43.2)
≥50 years	58 (56.8)
Menopausal status	
Yes	56 (54.9)
No	46 (45.1)
Mammographic findings	
Nodules	100 (98)
Microcalcifications	2 (2)
Molecular classification (immunohistochemistry)	
Luminal	19 (18.8)
Luminal/HER2	2 (2.0)
HER2-amplified	6 (5.9)
Triple-negative	74 (73.3)
Not available	1
Association with invasive ductal carcinoma, invasive lobular carcinoma, or ductal carcinoma in situ	
Yes	37 (36.3)
No	65 (63.7)
Clinical stage (*)	
I	20 (20)
II	21 (21)
III	59 (59)
Not available	2
Clinical size (cT)	
T1	4 (4)
T2	28 (28)
T3	26 (26)
T4b	30 (30)
T4d	12 (12)
Not available	2
Clinical lymph node status (cN)	
N0	49 (49)
N ≥ 1	51 (51)
Not available	2
Histological grade	
1	0
2	4 (4.5)
3	85 (95.5)
Not available	13
Pathologic stage (*)	
I	27 (27.8)
II	30 (30.9)
III	40 (41.2)
Not available	5
Pathological size (pT)	
T0	4 (4.0)
T1	13 (12.9)
T2	36 (35.6)
T3	27 (26.7)
T4b	19 (18.8)
T4d	2 (2.0)
Not available	1
Lymph node pathologic stage (pN)	
N0/N0(i+)	55 (55)
N1	25 (25)
N2	10 (10)
N3	10 (10)
Not available	2
Metaplastic breast carcinoma subtype	
Adenosquamous	10 (11)
Squamous cell	29 (31.8)
Spindle cell	6 (6.6)
Mesenchymal differentiation	26 (28.6)
Mixed	20 (22)
Unspecified	11
Not determined	

(*) Clinical and pathological stages were described in accordance with the AJCC – Cancer Staging Manual version at the time of diagnosis.

**Figure 1:**
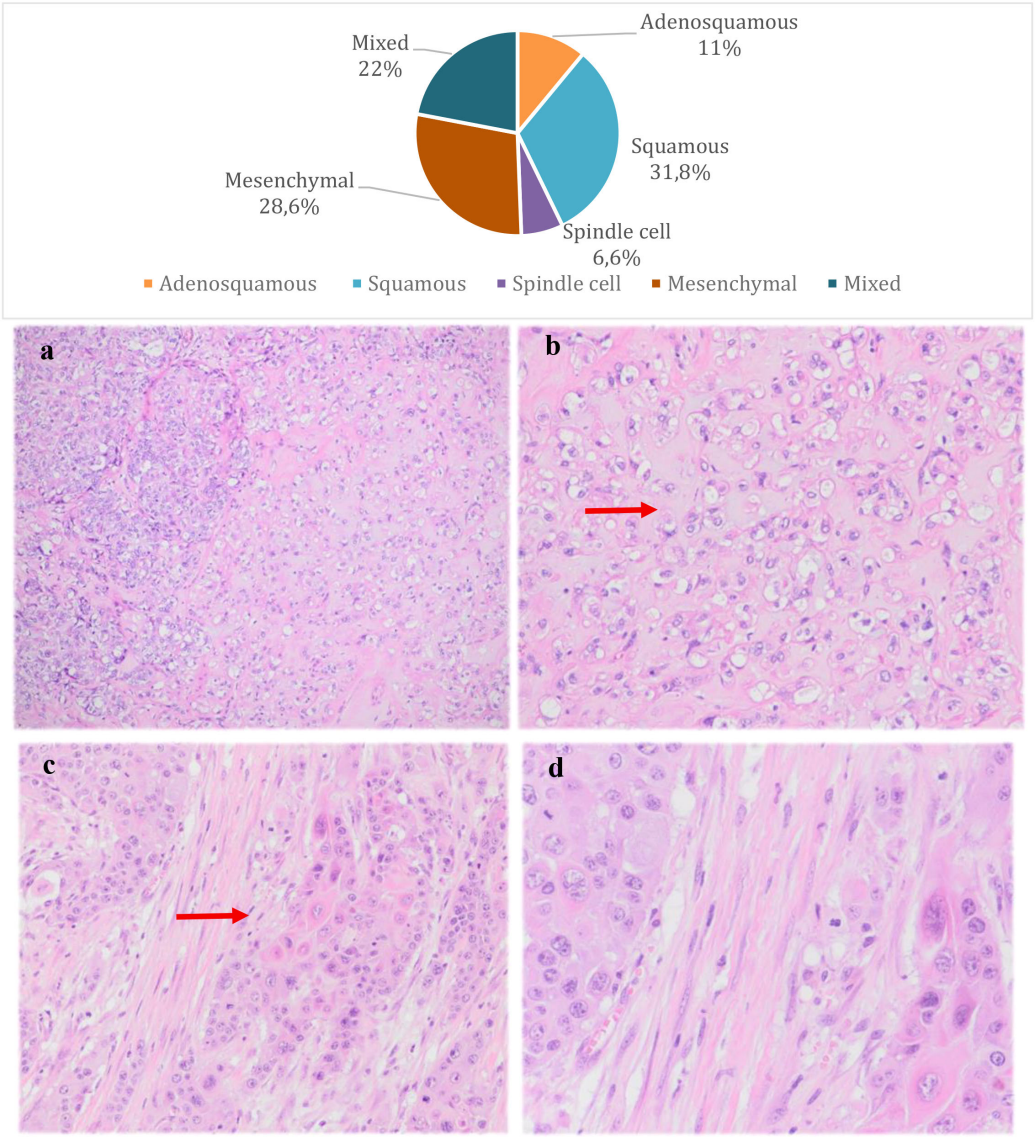
Distribution of metaplastic breast carcinoma subtypes and pathological features. Microphotographs of the pathological features of the most common subtypes of MBC; **(a)** High-grade invasive carcinoma with focal areas of chondroid differentiation (mesenchymal MBC) (H&E, 10x); **(b)** Invasive carcinoma with chondroid differentiation (mesenchymal MBC) (H&E, 40x); **(c)** Invasive metaplastic carcinoma showing a focus of dyskeratosis compatible with squamous differentiation (squamous MBC) (H&E, 20x); **(d)** Invasive metaplastic carcinoma showing a focus of dyskeratosis compatible with squamous differentiation (squamous MBC) (H&E, 40x);.

The most frequent subtypes of MBC, according to WHO classification (2019), were tumors with squamous and mesenchymal differentiation (sarcomatous, cartilaginous or osseous), in 31.8% and 28.6% of cases, respectively. In 22% of cases, the differentiation of MBC was mixed (**Figure 1**). It is worth mentioning that in 13 cases the subtype of MBC was not specified in the histopathology report and in 63.7% of the cases there was no association with other forms of breast carcinomas (IDC, ILC or DCIS) on histopathology.

**Table 2** describes the frequencies of the treatment modality provided. The mean time between patient admission to the oncology center and the first treatment was about 1.8 months. The initial treatment of the disease was neoadjuvant chemotherapy in around 52.9% of the cases, and the remaining women underwent to up front surgery. From the 54 patients underwent neoadjuvant chemotherapy, 14 (25.9%) had clinical progression, 19 (35.2%) had stable clinical disease, 19 (35.2%) had a partial clinical response and two (3.7%) had a complete clinical response. Only 30 of them were evaluated by RCB (*Residual Cancer Burden*) on postoperative histopathology and 86.6% (26/30) showed RCB II or III, which indicated moderate to extensive residual tumor burden after neoadjuvant therapy. The remaining four patients evaluated had RCB 0 (pathological complete response). The pCR rate was 7.4% (4/54) and the MBC subtypes were: one case with mesenchymal differentiation, one with spindle cell differentiation, one with squamous differentiation and one without a specific subtype (three of these tumors were triple-negative and one luminal/Her-2).

**Table 2:** Description of treatment approaches to metaplastic breast carcinoma.

Item	*N* = 102 (%)
Neoadjuvant chemotherapy	
Yes	54 (52.9)
No	48 (47.1)
Breast surgery	
Mastectomy	84 (82.3)
Breast-conserving surgery	18 (17.6)
Axillary surgery	
Sentinel lymph node dissection	27 (26.7)
Axillary dissection	74 (73.3)
Not available	1
Adjuvant chemotherapy	
Yes	47 (46.1)
No	55 (53.9)
Adjuvant radiotherapy	
Yes	81 (79.4)
No	21 (20.6)
Adjuvant endocrine therapy	
Yes	20 (19.6)
No	82 (80.4)
Clinical response to neoadjuvant therapy (N= 54)	
Complete remission	2 (3.7)
Partial remission	19 (35.2)
Stable disease	19 (35.2)
Progression	14 (25.9)
Residual cancer burden index	
0	4 (13.3)
II	13 (43.3)
III	13 (43.3)
Not available	24

Concerning surgical treatment, 82.3% of the patients underwent mastectomy and only 17.6% underwent breast-conserving surgery. Immediate breast reconstruction with a breast implant was performed in only 18 out of 84 patients undergoing mastectomy. Axillary lymph node dissection was performed in 73.3% of the cases and sentinel lymph node investigation was done in only 26.7% (**Table 2**). The average number of lymph nodes removed in axillary surgeries and those affected by disease was 15 and 2.8, respectively (data not shown).

Adjuvant chemotherapy was performed in 46.1% of the cases, that included anti-HER2-targeted therapy. The regimens were mainly based on anthracyclines and/or taxanes (85.1%) (data not shown). Of the 47 patients who received adjuvant chemotherapy, nine stage III patients also underwent neoadjuvant therapy, although three of these had showed disease progression during neoadjuvant therapy and only two patients had completed the neoadjuvant therapy cycles proposed. Six of those received additional systemic treatment during adjuvant therapy (with anthracyclins and/or taxanes or CMF) and another three patients received only anti-Her-2 therapy (**Supplementary Table 2**). Only 19.6% of the patients underwent endocrine therapy with tamoxifen or aromatase inhibitor. Adjuvant radiotherapy was performed in 80.4% of the patient population.

### 3.1 Disease-free survival and overall survival rates

**Figure 2:**
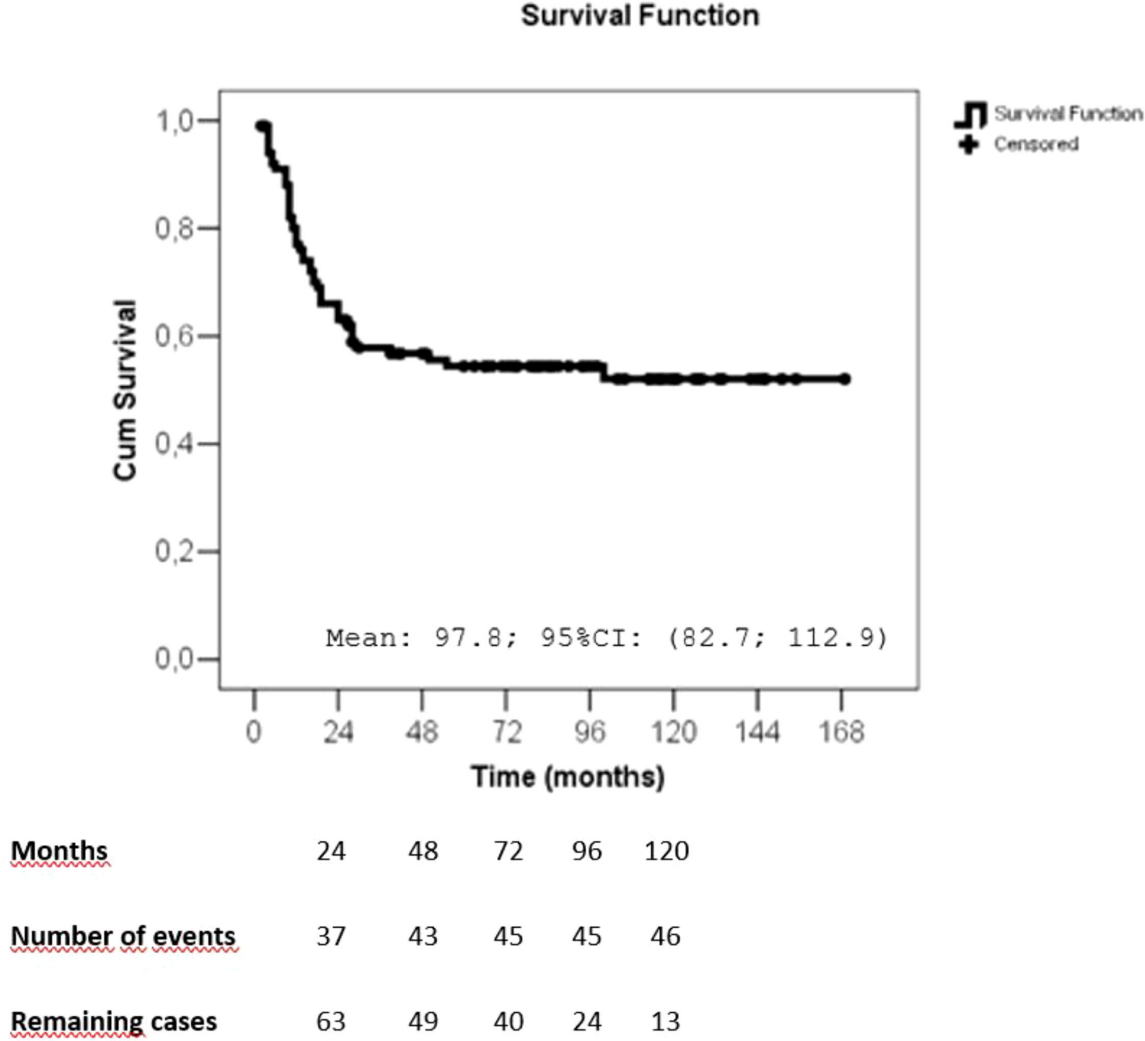
Disease-free survival (months) of patients with metaplastic breast cancer, as estimated by the Kaplan–Meier method. CI, confidence interval.

**Figure 3:**
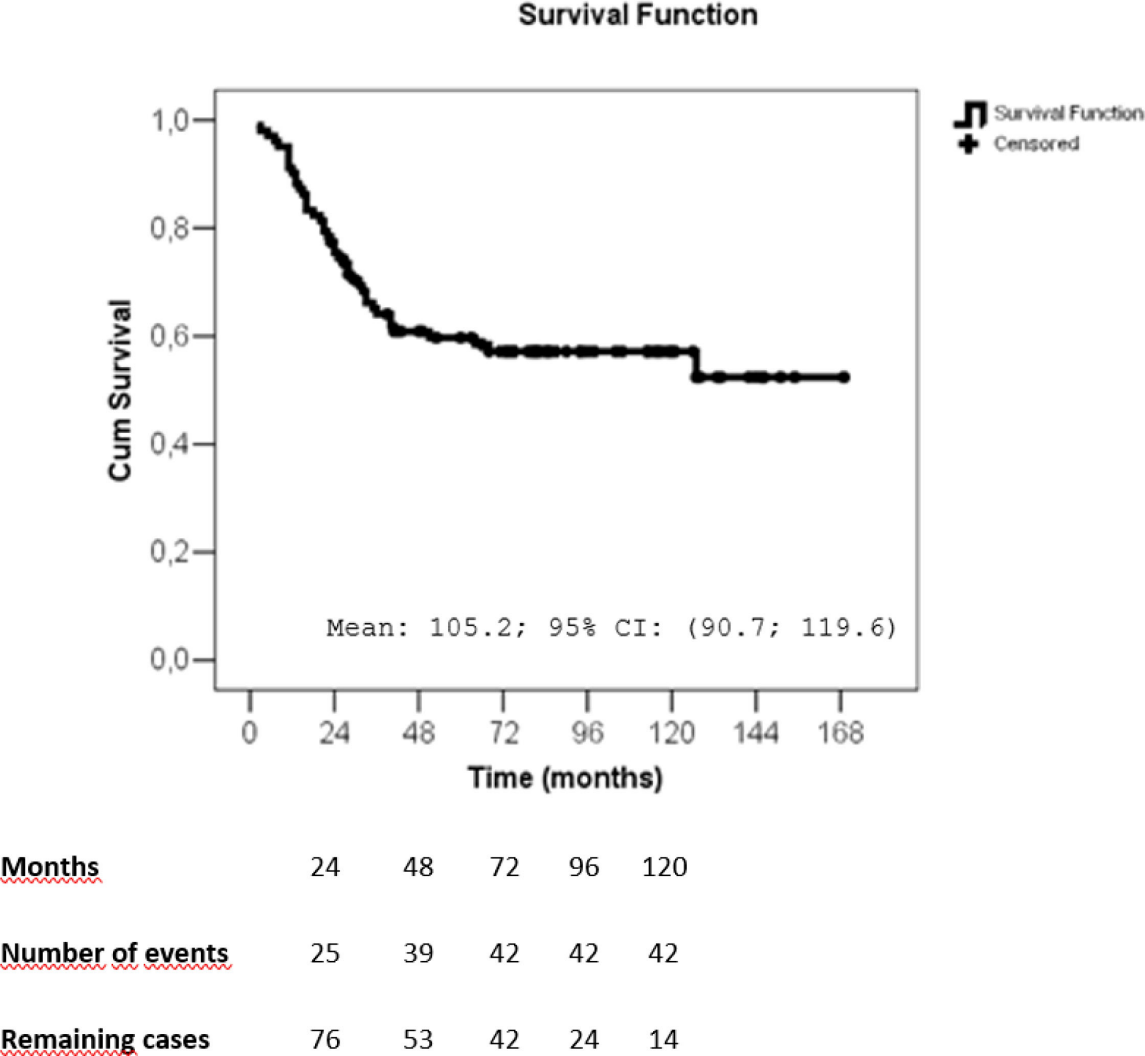
Overall survival of patients with metaplastic breast cancer, as estimated by the Kaplan–Meier method. SE, standard error; CI, confidence interval.

The mean follow-up period was 62.8 months. Recurrences occurred in 46 patients (45.1%), with 7 isolated locoregional recurrences, 32 distant recurrences and 7 both (locoregional and distant recurrences). Forty-three deaths were observed (42.1%) and 67.4% of the cases had breast cancer as the confirmed cause (**Table 3**). The disease-free survival curve (Kaplan-Meier) showed that the most recurrences occurred in the first 24 months, stabilizing in approximately 50% after 36 months (**Figure 2**). The overall survival curve (Kaplan-Meier) showed that the most deaths occurred in the first 36 months, stabilizing afterwards (**Figure 3**).

**Table 3:** Recurrences and deaths in follow-up.

Number of patients (%)	Number of (%)
**Recurrences**	**46 (45,1) recurrences**
Isolated locoregional	7 (15,2)
Distant recurrences	32 (69,6%)
Locoregional and distant recurrences	7 (15,2)
**Deaths**	**43 (42,1) deaths**
Breast cancer related cause	29 (67,4)
No information	14 (32,6)

Bold values indicates the number of patients with recurrences and the number of deaths.

**Figure 4:**
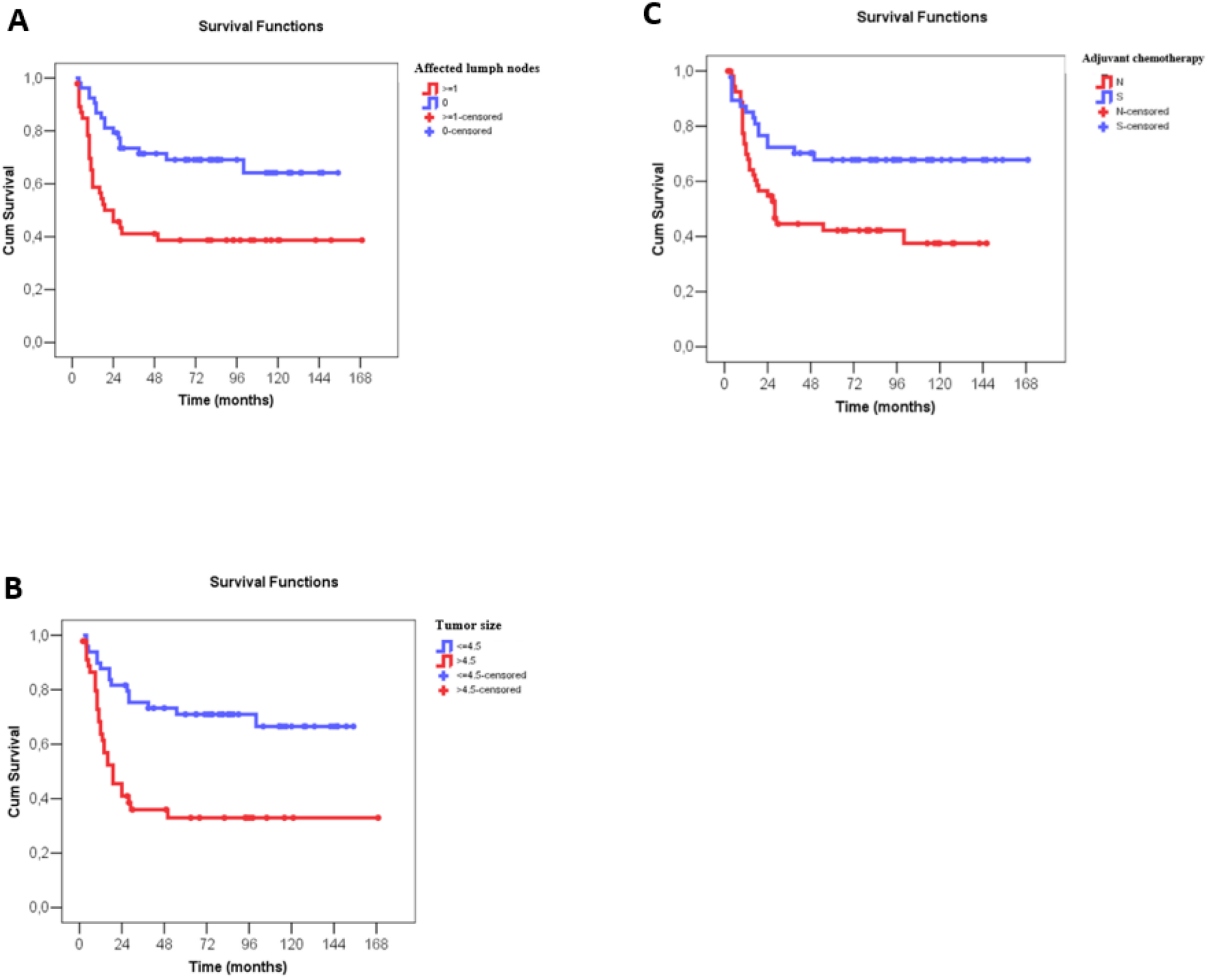
Disease-free survival (months) of patients with metaplastic breast câncer according to number of affected lymph nodes, tumor size at surgery and use of adjuvant chemotherapy. **(A)** Disease-free survival (months) according to number of affected lymph nodes, with N0 indicating no lymph node involvement and N ≥ 1 indicating metastatic involvement of lymph nodes (*). **(B)** Disease-free survival (months) according to tumor size (cm), as described in the surgical pathology report, classified as ≤4.5 cm and >4.5 cm (*). **(C)** Disease-free survival (months) according to administration or not of adjuvant chemotherapy. Numerical variables were divided by the median value in survival curve analyses.

The Cox univariate analyses showed worse disease-free survival for larger clinical and pathological tumor size, nodal status, higher clinical and pathological staging, administration of neoadjuvant chemotherapy, immediate breast reconstruction, larger number of metastatic lymph nodes and no administration of adjuvant chemotherapy. Multivariate analysis showed that only a higher number of metastatic lymph nodes, larger tumor size on surgical pathology and no administration of adjuvant chemotherapy were associated negatively with disease-free survival (**Supplementary Table 3** and **Table 4**). For each affected lymph node, the recurrence risk increased by 6.5% and for each additional centimeter of tumor size, on surgical pathology, the recurrence risk increased by 17.9%. A three-fold increase in the recurrence risk was observed, when adjuvant chemotherapy was not performed (**Figure 4**).

**Table 4:** Cox regression analysis results for disease-free survival in patients with metaplastic breast carcinoma according to number of affected lymph nodes, surgical size, and use of adjuvant therapy (*n* = 90).

Variable	Category	p-value	HR	95% CI
Number of affected lymph nodes	Continuous variable	**0.003**	1.065	1.022–1.109
Surgical size	Continuous variable (cm)	**<0.001**	1.179	1.092–1.274
Adjuvant chemotherapy	Yes (reference)	—	1.00	—
	No	**0.002**	3.00	1.52–5.93

HR, recurrence hazard ratio (*n*=47 censored, *n*=43 recurrences); CI, confidence interval; Bold values indicates p-value (statistical significance of the association). A stepwise variable selection procedure was used.

**Figure 5:**
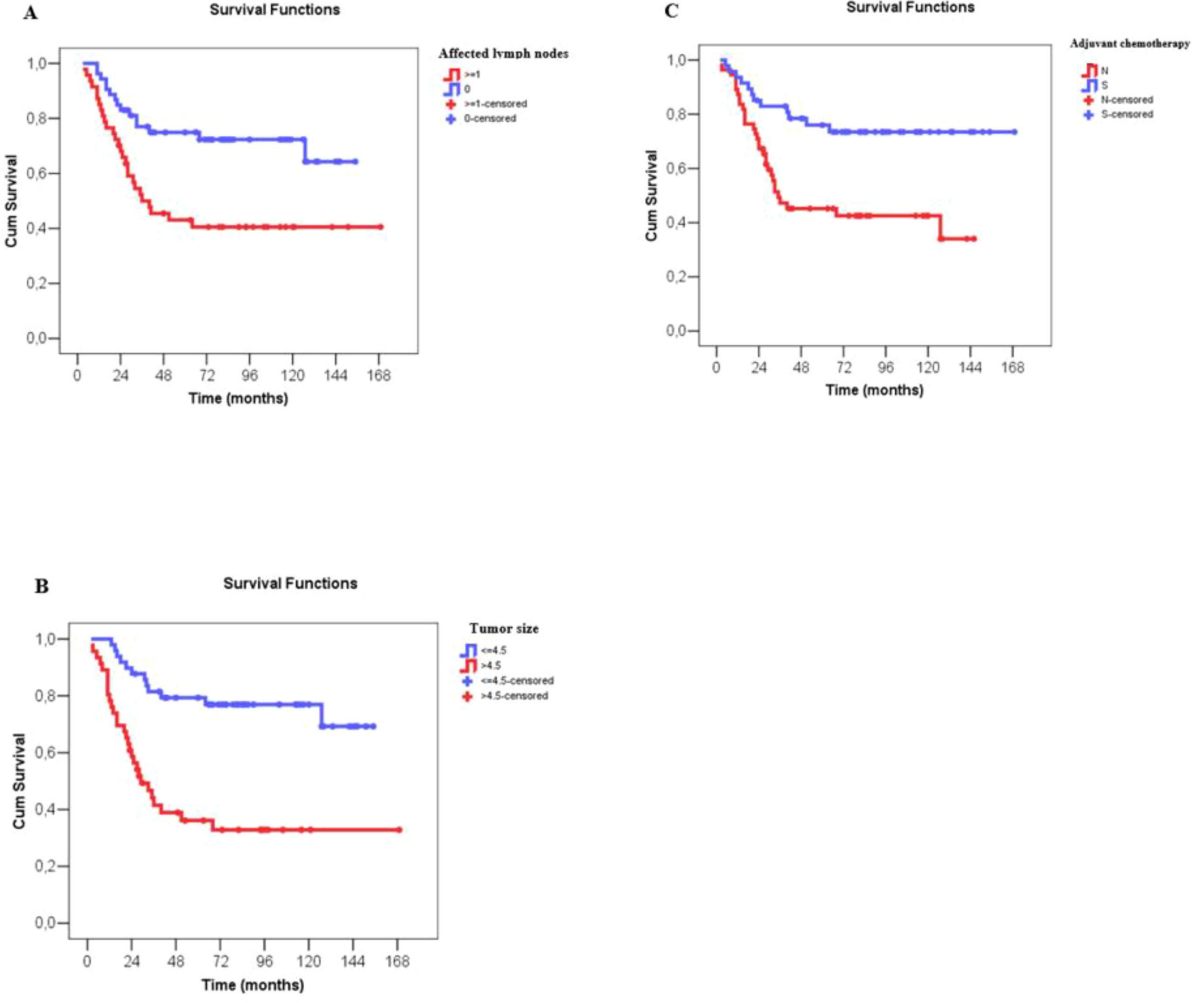
Overall survival (months) of patients with metaplastic breast câncer according to number of affected lymph nodes, tumor size at surgery and use of adjuvant chemotherapy. **(A)** Overall survival (months) according to number of affected lymph nodes, with N0 indicating no lymph node involvement and N ≥ 1 indicating metastatic involvement of lymph nodes (*). **(B)** Overall survival (months) according to tumor size (cm), as described in the surgical pathology report, classified as ≤4.5 cm and >4.5 cm (*). **(C)** Overall survival (months) according to administration or not of adjuvant chemotherapy. Numerical variables were divided by the median value in survival curve analyses.

The Cox univariate analyses showed worse overall survival for larger clinical and pathological tumor size, nodal status, higher clinical and pathological staging, administration of neoadjuvant chemotherapy, type of surgery on the breast, larger number of metastatic lymph nodes and no adjuvant chemotherapy. Similar to disease-free survival, multivariate analysis showed that only a higher number of affected lymph nodes, larger tumor size on surgical pathology and no administration of adjuvant chemotherapy associated negatively with overall survival (**Supplementary Table 4** and **Table 5**). For each affected lymph node, the risk of death increased by 8.6%, and for each additional centimeter of tumor size, on surgical pathology, the risk of death increased by 17.9%. No administration of adjuvant chemotherapy increased the risk of death in 3.9 times (**Figure 5**).

**Table 5:** Cox regression analysis results for overall survival in patients with metaplastic breast carcinoma according to number of affected lymph nodes, surgical size, and use of adjuvant therapy (*n* = 90).

Variable	Category	*p*-value	HR	95% CI
Number of affected lymph nodes	Continuous variable	**<0.001**	1.086	1.047–1.136
Surgical size	Continuous variable (cm)	**<0.001**	1.179	1.094–1.270
Adjuvant chemotherapy	Yes (reference)	—	1.00	—
	No	**<0.001**	3.86	1.84–8.09

Mortality hazard ratio (*n*=50 censored, *n*=40 deaths); CI, confidence interval; Bold values indicates p-value (statistical significance of the association). A stepwise variable selection procedure was used.

## 4 Discussion

MBC are breast malignancies with different morphological subtypes and the frequency of these seems to vary in different populations already studied. In our study, MBC with squamous differentiation was the most common subtype of tumor (32%), followed by mesenchymal (28.6%) and mixed cell (22%). Zhang et al. (2015), studying the Chinese population, observed a higher rate of MBC with spindle-cell differentiation (34.4%), while Cimino-Mathews et al. (2016), in a study at Johns Hopkins Hospital, found higher incidences of MBC with chondroid (24%) and mixed (28%) differentiation (18, 19). In a multi-institutional case series (2015) with 364 cases, a higher incidence of the squamous subtype (34%) was observed in Asian centers and a higher incidence of spindle cell tumors (34%) was found in European centers (20). Some studies have reported that the subtype of MBC was not associated with oncological outcome. Nevertheless, Hu et al. (2023) described that the mixed subtype could be related to a worse prognosis than MBC of a single morphological subtype (21, 22). In our analysis, the subtype of MBC was not associated with DFS or OS.

The concomitant occurrence of MBC with other forms of breast carcinoma varies from 57% to 73% in the literature (19, 20). In our series, we observed an association rate of 36.3%, with no impact on DFS or OS. On the other hand, Corso et al. (2021) observed lower rate of MBC with other forms of carcinomas (21.8%), however this was associated with worse OS (12). Tumor size in clinical staging is a parameter for surgical planning, indication of adjuvant therapies and disease prognosis. In this analysis, we found that T4 tumors at the time of clinical staging occurred in 42% of cases and the mean tumor size was 7.4 cm. A study in Cleveland Clinic Foundation – USA with 113 MBC patients showed that 60% had T2 tumors, and with average tumor size of 3.0 cm (23). Other studies have described similar data, showing T2 tumor size staging as the most common among MBC. The more aggressive phenotype of MBC and probably some socioeconomic barriers might explain the larger tumor size in our sample.

Rates of axillary lymph node metastasis in MBC seem being lower than ductal carcinoma breast cancer, ranging up to 30% in some studies (24–27). In our study, 51% of the patients had clinical lymph node disease (cN+) and 45% showed some lymph node disease burden on the surgical pathology, that could be considered low rates, taking into account that more advanced size of these tumors.

Some predictors of a poor prognosis in MBC have been described, as follows: large tumor, presence of lymph node metastasis, poorly differentiated tumor, young age (under 40 years) at diagnosis and skin invasion (3, 10, 19, 24). In this study, DFS and OS rates were associated with the variables related to advanced disease as larger tumor size and lymph node involvement.

MBC are frequently triple-negative tumors and the rate of tumors with positive hormonal receptors (HR) varies from 5% to 20% and with Her-2 amplified from 0 to 16% (28). In previous studies, there is no apparent association between a better prognosis of MBC with positive HR, although this positivity is historically related to better survival in other histological subtypes of breast carcinoma (29–32). In the present study, 73.2% of the patients was classified as triple-negative, 18.8% as luminal, 1.98% as luminal/Her2 and 5.9% as Her-2 amplified. These data are in agreement with previous studies on molecular subtypes assessed by immunohistochemistry among MBC patients (31, 33).

According to current guidelines for breast cancer treatment, such as the NCCN (National Comprehensive Cancer Network), in cases of locally advanced disease or triple-negative and Her-2 positive tumors, even in early stages, neoadjuvant systemic therapy is recommended (34). However, although MBC is usually triple-negative and often locally advanced, chemoresistance is described in previous data and shows that neoadjuvant chemotherapy may be associated with worse oncological outcomes in these tumors (3, 35). Pathological complete response (PCR) occurs in around 0% to 28% of cases and disease progression during neoadjuvant treatment varies from about 5% to 50% (3, 29, 35–37). Of the 54 patients undergoing neoadjuvant treatment in our sample, we observed that clinical disease progression occurred in 25.9% of patients and only 7.4% (4 out of 54) achieved pCR on surgical pathology, demonstrating that the effectiveness of this treatment modality for MBC is low and negatively influences in disease prognosis.

With recent data from the Keynote-522 trial, Pembrolizumab has become the gold standard immunotherapy drug for treatment of TN tumors in neoadjuvant therapy. However, only reports based on case series of metastatic MBC showed favorable response to the use of Pembrolizumab or Atezolizumab (15, 38–40).

In our population, the 5-year DFS among patients undergoing adjuvant chemotherapy and those who did not receive the treatment was 68.8% and 42.2%, respectively (p=0.009). Similar data for 5-year OS rate showed 76% and 45.2%, respectively (p=0.001). Although Cox regression multiple analyses showed better association of adjuvant therapy and prognosis, most probably there were biases because of the study design was not appropriated for this comparison, even though the findings of the neoadjuvant chemotherapy and adjuvant chemotherapy were clinically discrepant. In the literature, the relationship between adjuvant chemotherapy and survival in MBC is divergent. Some studies showed an association between the administration of chemotherapy and better OS rates (Cecilia T. Ong et al., Meng Xiao et al., Ashley Cimino-Mathews et al. and Min Han et al.), while in others this association was not statistically significant (So-Youn Jung et al., Hyewon Lee et al., Yiqian Zhang et al. and Xuexin He et al.) (14, 18, 19, 36, 41–44).

A higher rate of surgical treatment with mastectomy in MBC patients has been observed in the literature (ranging from 36% to 92%). The large number of mastectomies may be related to disease aggressiveness, larger tumor size at diagnosis and a low response to neoadjuvant therapy (45, 46). Similar to previous data, mastectomy was performed in 82.5% (84/102) of the cases and only a minority of patients underwent immediate breast reconstruction (18/84). There is no previous data on breast reconstruction in the scenario of MBC treatment. In our analysis, the recurrence rates among reconstructed and non-reconstructed patients were 8.7% x 91.3%, respectively (p=0.032). This data is probably associated with a careful selection of patients eligible for immediate breast reconstruction.

Adjuvant radiotherapy was carried out in around 79.4% of the patients, but no impact was observed on DFS or OS survivals. In this context, Tseng et al. demonstrated improvement in OS and cancer-related survival in MBC patients who underwent adjuvant radiotherapy, regardless of the type of surgical treatment performed (47). In other studies, there was an improvement in OS with the association of adjuvant radiotherapy only in cases of locally advanced disease and intermediate risk for recurrence after mastectomy (14, 33, 48, 49). On the other hand, radiotherapy was not significantly associated with improved DFS in part of the studies with this evaluation (18, 36, 42, 43). Therefore, comparisons between these findings are not appropriate due to the heterogeneity between study designs.

MBCs are tumors with a high risk for recurrences and this usually occur in the first years of follow-up after treatment (42). We observed a 5-year DSF rate of 54.4% and a 5-year OS rate of 59.7%, with 46 recurrences. Of these, 84.8% (39/46) had distant recurrences and only 15.2% (7/46) had isolated locoregional recurrence, indicating that MBC has a higher chance of metastasizing. The 5-year DFS and OS rates in this evaluation were similar to those found in the literature, in which DFS ranged from 30% to 81.5% and OS from 54% to 93%. Elimimian et al. (2021) revealed that the 5-year OS for TN MBC was 63.1%, worse rates than in other types of triple-negative breast cancers, in addition to a higher tendency for metastasis (50, 51).

A limitation of the study was, obviously, its retrospective design that revealed unappropriated for some analyses, mainly for evaluating the outcome according to the treatment modalities. The treatment guidelines of each institution were similar, but it was not the same. Nevertheless, this study included a large case series of a rare tumor, assisted in two Brazilian oncology centers, that offer data and knowledge to better understand this heterogeneous disease.

## 5 Conclusion

According to results of this study, MBC presented as a large nodular lesion at diagnosis, the most frequent metaplastic subtypes presented squamous and mesenchymal differentiation, almost 80% were triple-negative tumors, however, responses to neoadjuvant chemotherapy can be considered poor. Higher disease progression rates were observed in MBC during neoadjuvant therapy and the complete pathological response rates were lower. Among the variables analyzed by multivariate Cox regression, higher number of metastatic lymph nodes and larger tumor size were associated with worse DFS and OS, meanwhile the women who undergone to adjuvant chemotherapy showed better DFS and OS. Furthermore, most recurrences occurred in the first 24 months of follow-up, stabilizing at approximately 50% after 36 months, and most deaths occurred in the first 36 months, stabilizing thereafter, which is a clinical pattern of very aggressive tumors.

## Data Availability

The original contributions presented in the study are included in the article/**Supplementary Material**. Further inquiries can be directed to the corresponding author.

## Supplementary material

Supplementary Table 1 Descriptive statistics of the numerical variables analyzed in the study.

Supplementary Table 2 Cases given neoadjuvant and adjuvant therapy.

Supplementary Table 3 Results of Cox univariate regression analysis for disease-free survival (n=102).

Supplementary Table 4 Results of Cox univariate regression analysis for overall survival (n=102).
