# Supplementary material for: Clinicopathological characteristics, treatments and oncological outcomes in metaplastic breast cancer: a Brazilian multicenter analysis
Source: Front Oncol. 2025 Sep 29;15:1568178. doi: 10.3389/fonc.2025.1568178 (PMC12515624; doi:10.3389/fonc.2025.1568178)
Supplement: Supplementary Table 1 — Descriptive statistics of the numerical variables analyzed in the study. [file Table1.docx]

Supplementary Material

# Supplementary Data

***Table 1- Descriptive statistics of the numerical variables analyzed in the study.***

| **Item** | ***n*** | **Mean** | **Min** | **Max** | **Median** |
| --- | --- | --- | --- | --- | --- |
| Age (years) | 102 | 52.97 | 28 | 88 | 52 |
| Clinical tumor size (cm) | 97 | 7.44 | 1.2 | 30 | 6.0 |
| Ki-67 index (%) | 88 | 65.11 | 5 | 100 | 75 |
| Surgical tumor size (cm) | 95 | 6.01 | 0.3 | 20 | 4.5 |
| Time to treatment (months) | 99 | 1.78 | 1 | 6 | 1.0 |
| Number of affected lymph nodes | 100 | 2.8 | 0 | 30 | 0 |
| Follow-up (months) | 102 | 62.81 | 2 | 169 | 52 |

***Table 2- Cases given neoadjuvant and adjuvant therapy.***

| **N** | **Subtype molecular** | **Subtype MBC** | **CS** | **Neoadj.** | **CR** | **EP** | **Adjuvant** | **Event oncológico** | **Which?** |
| --- | --- | --- | --- | --- | --- | --- | --- | --- | --- |
| **1** | TPN | Mixed | III | 1xA + 3xT | Prog | III | 6xCMF | N | - |
| **2** | Luminal | Adenosquamous | III | 4xA | Prog | III | 4xT | S | RLR |
| **3** | Her-2 | Squamous | III | 4xA + 4xT | SD | III | H | S | RLR+RD |
| **4** | TPN | Adenosquamous | III | 4xA | SD | III | 4xT | N | - |
| **5** | TPN | Adenosquamous | III | 2xT | SD | III | 2xT | S | RLR |
| **6** | Luminal/Her-2 | Squamous | III | 4xA + 12xTH | Partial | RPC | H | S | NP |
| **7** | TPN | Squamous | III | 2xA | SD | II | 2xA + 12xT | N | - |
| **8** | Luminal | Squamous | III | 1xA | SD | III | 3xA + 10xT | N | - |
| **9** | Her-2 | Squamous | III | 3xA + 1xTH | Prog | III | H | S | RLR+RD |
| **Legend:** TPN-triple-negative; CS-clinical staging; CR-Clinical response; SD-stable disease, Prog-progression, pCR- pathological complete response; H-trastuzumab; A-anthracycline; T-taxane; CMF- cyclophosphamide/methotrexate/fluorouracil; N-não; S-sim; RLR- recidiva loco-regional; RD- recidiva a distância; NP- novo primário. | | | | | | | | | |

***Table 3- Results of Cox univariate regression analysis for disease-free survival (n=102).***

| **Variable** | **Categories** | | **P-value** | | **H.R.*** | | **95%CI H.R.*** | |  |
| --- | --- | --- | --- | --- | --- | --- | --- | --- | --- |
| **Age** | Continuous variable (years) | | 0.672 | | 1.005 | | 0.983 – 1.027 | |  |
| **Menopause** | No (ref.)  Yes | | ---  0.471 | | 1.00  0.81 | | ---  0.45 – 1.44 | |  |
| **Molecular subtype** | Triple-negative (ref.)  HER-2-amplified  Luminal  Luminal/HER-2 | | ---  0.435  0.701  0.212 | | 1.00  1.51  1.16  0.37 | | ---  0.54 – 4.27  0.55 – 2.42  0.03 – 4.74 | |  |
| **cT** | 1-2 (ref.)  3  4 | | ---  0.671  **<0.001** | | 1.00  1.22  3.80 | | ---  0.49 – 3.08  1.78 – 8.10 | |  |
| **cN** | 0 (ref.)  1  2-3 | | ---  0.926  **0.003** | | 1.00  1.03  2.86 | | ---  0.51 – 2.09  1.43 – 5.75 | |  |
| **Clinical staging** | I (ref.)  II  III | | ---  0.076  **0.008** | | 1.00  2.91  4.11 | | ---  0.89 – 9.45  1.45 – 11.63 | |  |
| **Neoadjuvant chemotherapy** | No (ref.)  Yes | | ---  **0.026** | | 1.00  2.02 | | ---  1.09 – 3.75 | |  |
| **Breast surgery** | Quadrantectomy (ref.)  Mastectomy | | ---  0.078 | | 1.00  2.31 | | ---  0.91 – 5.84 | |  |
| **Axillary surgery** | PLS (ref.)  EA | | ---  0.077 | | 1.00  1.93 | | ---  0.93 – 4.01 | |  |
| **Admission time to 1st treatment** | Continuous variable (months) | | 0.911 | | 0.985 | | 0.753 – 1.288 | |  |
| **Subtype of MBC** | Adenosquamous (ref.)  Squamous  Spindle cell  Mesenchymal  Mixed  Without a specific type | | ---  0.553  0.188  0.516  0.678  0.793 | | 1.00  0.73  0.24  0.70  0.80  0.85 | | ---  0.26 – 2.07  0.03 – 2.03  0.25 – 2.03  0.27 – 2.33  0.25 – 2.93 | |  |
| **Association with IDC/ILC/DCIS** | No (ref.)  Yes | | ---  0.375 | | 1.00  1.30 | | ---  0.73 – 2.35 | |  |
| **Histological Grade** | | 2 (ref.)  3 | | ---  0.897 | | 1.00  1.10 | | ---  0.27 – 4.55 | |
| **Surgical size** | | Continuous variable (cm) | | **<0.001** | | 1.184 | | 1.108 – 1.265 | |
| **pT** | | 0-1 (ref.)  2  3  4 | | ---  0.677  **0.044**  **0.002** | | 1.00  1.25  2.84  4.94 | | ---  0.44 – 3.61  1.03 – 7.86  1.78 – 13.68 | |
| **pN** | | 0 (ref.)  1  2  3 | | ---  0.184  **<0.001**  **<0.001** | | 1.00  1.67  6.14  6.34 | | ---  0.78 – 3.57  2.68 – 14.08  2.70 – 14.91 | |
| **Number of lymph nodes affected** | | Continuous variable | | **<0.001** | | 1.111 | | 1.070 – 1.154 | |
| **Pathological staging** | | I (ref.)  II  III | | ---  0.093  **<0.001** | | 1.00  2.32  6.16 | | ---  0.87 – 6.19  2.53 – 14.98 | |
| **Adjuvant radiotherapy** | | No (ref.)  Sim | | ---  0.575 | | 1.00  0.81 | | ---  0.39 – 1.68 | |
| **Adjuvant chemotherapy** | | Yes (ref.)  No | | ---  **0.012** | | 1.00  2.22 | | ---  1.19 – 4.12 | |
| **Adjuvant endocrine therapy** | | No (ref.)  Yes | | ---  0.234 | | 1.00  0.61 | | ---  0.27 – 1.37 | |
| * HR (*Hazard Ratio*) = Hazard ratio for recurrence; (n=56 Censors and n=46 Recurrences). 95%CI HR = 95% confidence interval for hazard ratio. Ref.:level of reference. | | | | | | | | | |

***Table 4- Results of Cox univariate regression analysis for overall survival (n=102).***

| **Variable** | **Categories** | **P-value** | **H.R.*** | **95% CI H.R.*** |
| --- | --- | --- | --- | --- |
| **Age** | Continuous variable (anos) | 0.661 | 1.005 | 0.983 – 1.028 |
| **Menopausal** | No (ref.)  Sim | ---  0.447 | 1.00  0.79 | ---  0.44 – 1.44 |
| **Molecular subtype molecular** | TPN (ref.)  HER2+  Luminal  Luminal/HER2 | ---  0.490  0.877  0.237 | 1.00  1.44  1.06  0.40 | ---  0.51 – 4.10  0.49 – 2.32  0.03 – 5.05 |
| **cT** | 1-2 (ref.)  3  4 | ---  0.375  **<0.001** | 1.00  1.62  5.43 | ---  0.56 – 4.67  2.24 – 13.14 |
| **cN** | 0 (ref.)  1  2-3 | ---  0.327  **0.004** | 1.00  1.44  2.96 | ---  0.70 – 2.99  1.42 – 6.15 |
| **Clinical Staging** | I (ref.)  II  III | ---  **0.001**  **<0.001** | 1.00  18.14  24.15 | ---  1.12 – 292.41  1.55 – 376.73 |
| **Neoadjuvant chemotherapy** | No (ref.)  Sim | ---  **0.024** | 1.00  2.09 | ---  1.10 – 3.97 |
| **Breast surgery** | Quadrantectomy (ref.)  Mastectomia | ---  **0.015** | 1.00  11.70 | ---  1.61 – 85.07 |
| **Axillary surgery** | PLS (ref.)  EA | ---  **0.012** | 1.00  3.34 | ---  1.31 – 8.50 |
| **Admission to 1st treatment treatment** | Continuous variable (months) | 0.954 | 0.992 | 0.746 – 1.318 |
| **Subtype of MBC** | Adenosquamou (ref.)  Squamous  Fusocelular  Mesenquimal  Misto  Sem especificação | ---  0.442  0.548  0.581  0.774  0.549 | 1.00  1.64  0.50  1.43  1.22  1.55 | ---  0.47 – 5.75  0.05 – 4.80  0.40 – 5.14  0.32 – 4.58  0.37 – 6.49 |
| **Association with CDI/CLI/CDIS** | No (ref.)  Sim | ---  0.720 | 1.00  1.12 | ---  0.61 – 2.06 |
| **Histological grade** | 2 (ref.)  3 | ---  0.519 | 1.00  1.92 | ---  0.26 – 14.02 |
| **Surgical Size** | Continuous variable (cm) | **<0.001** | 1.187 | 1.111 – 1.267 |
| **pT** | 0-1 (ref.)  2  3  4 | ---  0.139  **0.008**  **0.002** | 1.00  3.13  7.52  10.59 | ---  0.69 – 14.14  1.71 – 33.07  2.41 – 46.50 |
| **pN** | 0 (ref.)  1  2  3 | ---  0.134  **<0.001**  **<0.001** | 1.00  1.81  4.60  6.48 | ---  0.83 – 3.96  1.92 – 11.03  2.71 – 15.51 |
| **Number of lymph nodes lymph nodes acometidos** | Continuous variable | **<0.001** | 1.137 | 1.088 – 1.188 |
| **Pathological staging patológico** | I (ref.)  II  III | ---  **0.006**  **<0.001** | 1.00  8.09  16.77 | ---  1.81 – 36.13  3.97 – 70.80 |
| **Adjuvant radiotherapy** | No (ref.)  Sim | ---  0.687 | 1.00  0.86 | ---  0.41 – 1.79 |
| **Adjuvant chemotherapy** | Yes (ref.)  Não | ---  **0.002** | 1.00  2.87 | ---  1.47 – 5.61 |
| **Endocrine therapy therEndocrinoterapia adjuvante** | No (ref.)  Sim | ---  0.102 | 1.00  0.46 | ---  0.18 – 1.17 |
| * HR (*Hazard Ratio*) = hazard ratio for death; (n=59 Censors and n=43 Deaths).95% CI HR = 95% confidence interval for hazard ratio Ref.: level of reference. | | | | |
